# High incidence of plasmids in marine *Vibrio* species isolated from Mai Po Nature Reserve of Hong Kong

**DOI:** 10.1007/s10646-012-0939-7

**Published:** 2012-06-09

**Authors:** Ruifu Zhang, Li Pan, Zhenye Zhao, Ji-Dong Gu

**Affiliations:** 1 Laboratory of Environmental Microbiology and Toxicology, School of Biological Sciences, The University of Hong Kong, Kadoorie Biological Sciences Building, Pokfulam Road, Hong Kong SAR, People’s Republic of China; 2 Jiangsu Key Lab for Organic Solid Waste Utilization, Nanjing Agricultural University, Nanjing, 210095 People’s Republic of China; 3 Shenzhen-Hong Kong Institution of Industry, Education, Research Environment Engineering Technique Co., Ltd, Shenzhen, Guangdong China; 4 The Swire Institute of Marine Science, The University of Hong Kong, Shek O, Cape d’Aguilar, Hong Kong SAR, People’s Republic of China

**Keywords:** *Vibrio cholerae*, Plasmid, Diversity, Distribution, Mai Po Nature Reserve, Replication

## Abstract

Mai Po Nature Reserve is the largest mangrove ecosystem and the most polluted coastal water body in Hong Kong. Plasmids screening of 100 *Vibrio* isolates randomly showed 45 % of them contained 1–3 plasmids. These plasmid(s)-bearing isolates could be divided into 12 groups based on their plasmid profiles. Phylogenetic analysis of the partial 16S rRNA gene sequences confirmed that all plasmid(s)-bearing isolates belonged to *Vibrio cholerae*. Full DNA sequences of the plasmids in Groups I (pVCG1.1 and pVCG1.2), II (pVCG2.1), III (pVCG3.2) and IV (pVCG4.1) have been determined and the results showed that pVCG1.1, pVCG2.1 and pVCG3.2 were almost identical. Plasmids pVCG1.1, pVCG1.2 and pVCG4.1 are comprised of 4,439, 2,357 and 2,163 bp with the overall G+C content of 45.57, 53.54 and 43.09 %, respectively. pVCG1.1 is a novel plasmid, and plasmids pVCG1.2 and pVCG4.1 showed homology of replication initiation proteins to that of the theta type replicons. Attempts to cure the plasmids from their hosts were unsuccessful. These data suggest that plasmids of *Vibrio* spp. are a significant gene reservoir in the marine ecosystem.

## Introduction

*Vibrio* species are natural inhabitants of aquatic environments, though many of the studies are primarily carried out on clinical strains, the environmental isolates may serve as a reservoir for the wide spread of antibiotic resistance or virulence genes due to horizontal gene transfer (Faruque et al. 1998; Chiang and Mekalanos 1999; Hazen et al. 2010). Plasmids and other mobile genetic elements such as prophages, integrons, genomic islands and transposons play vital and essential roles in the gene transfer processes. The high incidence of plasmid in bacteria of marine sediment suggested that marine environment is an important source for discovery of novel plasmids (Sobecky 1999, 2002; Hazen et al. 2010), but attributing specific traits and functions to the environmental plasmids have been proven difficult.

Of 440 isolates of presumptive marine *Vibrios* collected from the Gulf of Mexico, 31 % of them harbor plasmids, the plasmid incidence was 1.5-fold higher in oil field than in the control site located 8 km from the impacted sampling sites (Hada and Sizemore 1981). Although attempts have been made to assign phenotypic traits such as hydrocarbon utilization and heavy metal resistance to the plasmid(s)-bearing isolates, no correlation could be established between plasmid content and the resistance determinants. Davidson and Oliver (1986) examined 42 clinical and environmental isolates of *Vibrio vulnificus*, 5 (12 %) harbored plasmids, with various sizes, attempt to demonstrate a correlation between the presence of plasmids and a variety of phenotypic traits was unsuccessful, only the correlation between the presence of a 6.5-megadalton plasmid and the resistance to vibriostatic agent O/129 was observed. In another study, a relationship between high molecular weight plasmids and eel virulence was suggested by Biosca et al. (1997), who found isolates bearing high molecular weight plasmids showed lower LD_50_ in eels than those that are plasmid-free. Hoi et al. (1998) observed that 93 of 97 strains isolated from five outbreaks of *V. vulnificus* in Danish eel farms harbored from 1 to 3 high molecular weight plasmids, supporting the hypothesis of a possible participation of high molecular weight plasmids in virulence of eels.

In *Vibrio cholerae*, the presence of the conjugative P plasmid, which is not widely disseminated among clinical isolates, significantly attenuates pathogenicity due to plasmid-induced loss of virulence (Sinha and Srivastava 1978). Cook et al. (1984) investigated plasmid profiles of many clinical isolates of classical biotype *V. cholerae* strains, and found that classical strains possess two plasmids (the smaller one was about 4.7 kb). Bartowsky and Manning (1988) reported three plasmids in a *V. cholerae* classical strain V58: the P plasmid, a large cryptic plasmid (lcp, 34 kb), and a small cryptic plasmid (scp, 4.7 kb). However, little is known about these cryptic plasmids. Rubin et al. (1998) sequenced a 4.7 kb toxin-linked plasmid, which is identical to the smaller plasmid in the above-mentioned two studies. This plasmid has been designated as pTLC (toxin-linked cryptic). pTLC can exist as both extra-chromosomal double-stranded circular plasmid and tandem duplicated DNA, inserted on the chromosome at a position only 842 bp upstream of the CTX prophage. This plasmid may play a crucial role in the acquisition and replication of CTX prophage.

Systematic studies on plasmid distribution and diversity in marine ecosystems, particularly at the molecular level, should provide new insights and understanding of plasmid function. Mai Po Nature Reserve is the largest mangrove ecosystem and most polluted water body in Hong Kong. This nature reserve plays a very important role in supporting a wide range of wildlife including migratory birds, e.g., the endangered Black-faced Spoonbills with a global population of about 2,000 (Tsim and Lock 2002), of which more than 20 % have been recorded here. This protected area is also an important refueling station for winter birds on their flyway from Arctic and northern China to Australia. The Nature Reserve is threatened by increasing pollutions largely due to the economic development in the adjacent Shenzhen Special Economic Zone of the People’s Republic of China (Cao et al. 2011a, b; Li et al. 2011a, b, c; Shen et al. 2012; Zhao et al.  2012a, b). We have isolated and characterized two novel plasmids from *Vibrio* species in Mai Po Nature Reserve previously (Zhang et al. 2007; Zhang and Gu 2009; Pan et al. 2010). In this study, a systematic investigation on plasmids diversity and distribution of *Vibrio* species community was conducted.

## Materials and methods

### Sampling sites, *Vibrio* strain isolating, plasmid screening and grouping

**Fig. 1:**
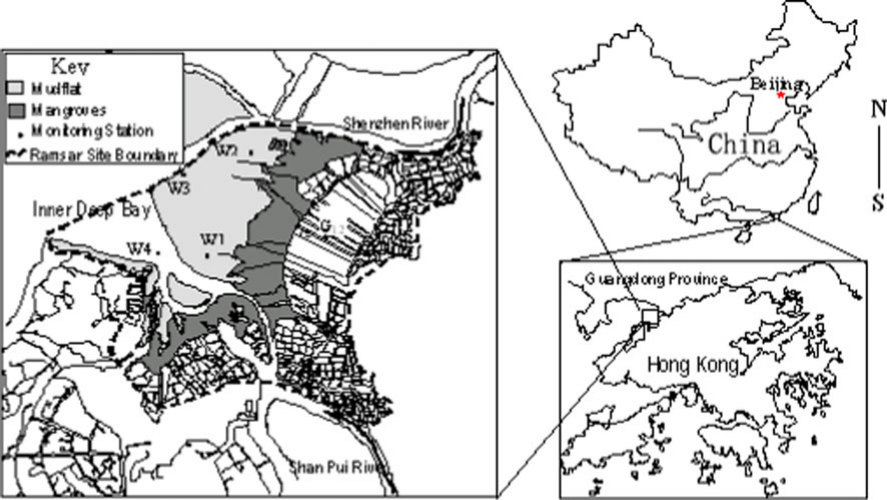
A map of the Mai Po Nature Reserve, Hong Kong showing the area with different habitats and sampling locations (W1–4, G12)

Water samples were collected from several previously determined sampling sites (W1, W2, W3, W4 and G12) at Mai Po Nature Reserve (22°29′N to 22°31′N and 113°59′E to 114°03′E) of Hong Kong SAR, PR China (Fig. 1). Surface water was taken to fill 1 L sterile plastic bottles when the tidal level was at approximately 1.5 m at this site. All samples were kept in coolers and transported immediately back to the laboratory after sampling for further processing.

Samples for this study were collected in July 2005. At the time of sampling, in situ temperature, pH, dissolved oxygen, salinity, turbidity and suspended solids were 31.2–32.9 °C, 6.51–7.59, 6.82–13.09 mg/L, 2.2–4.6 ‰, 25.9–84.3 mg/L and 45–158 NTU, respectively. Chemical analysis of the water samples showed that NH_4_^+^-N, NO_3_^−^-N, NO_2_^−^-N, total Kjeldahl N, *ortho*-P, total P and BOD_5_ was 3.28–5.52, 0.05–0.27, 0.18–0.33, 1.77–3.24, 0.45–0.67, 0.51–0.82, and 6.12–53.2 mg/L, respectively.

Water samples (0.1 mL) were directly spread onto TCBS agar plates and incubated at 30 °C for about 12 h. Colonies on agar plates were picked and streaked on new TCBS agar plates several times to obtain pure cultures of the isolates. One hundred purified isolates were randomly selected for screening of plasmids. Plasmid screening was carried out using alkaline lysis technique as described by Sambrook et al. (1989). Plasmids for further research were prepared with Qiaprep spin miniprep kit (Qiagen Inc, Valencia, California, USA) for plasmids less than 10 kb, or with alkaline lysis technique for plasmids greater than 10 kb. Each plasmid was separately purified through gel extraction, followed by column purification or ethanol precipitation. Plasmid(s)-bearing isolates were grouped based on their plasmid profiles in terms of the number of plasmid, plasmid size and their RFLP analyses using the enzymes of *Eco*RI and *Kpn*I.

### Bacterial strains, plasmids and molecular genetic techniques

All *Vibrio* isolates were obtained from the water samples of Mai Po Nature Reserve, Hong Kong. *E. coli* JM109 cells and pUC19 cloning vector were from laboratory storage. Restriction analysis, DNA ligation and transformation were performed according to standard protocols (Sambrook et al. 1989).

### Plasmids sequencing and analyses

Plasmids pVCG1.1, pVCG1.2, pVCG2.1 and pVCG3.2 have a single *EcoR*I site and pVCG4.1 has a *BamH*I site, which were used to clone them into *EcoR*I or *BamH*I digested pUC19 vector, recombinant plasmids carrying the linear plasmids fragments were designated as pUVG1.1, pUVG1.2, pUVG2.1, pUVG3.2 and pUVG4.1, respectively. The inserted linear plasmids sequences of pUVG1.1, pUVG1.2, pUVG2.1, pUVG3.2 and pUVG4.1 were obtained using pUC19 vector specific primers, and then the internal primers derived from the sequence information through the previous sequencing cycle. Sequences of both strands were determined at Department of Zoology, The University of Hong Kong.

DNA sequence analysis were performed using Bioedit (Hall 1999). Database searches were carried out using BLASTn and BLASTp (Altschul et al. 1997), putative ORFs were determined using both the online ORF finder software on the NCBI website and GeneMark software (http://opal.biology.gatech.edu/GeneMark/).

### Phylogenetic analyses

16S rRNA genes were amplified using the universal primer 8F (5′-AGAGTTTGATCCTGGCTCAG-3′; *E. coli* bases 8–27) and reverse primer 1492R (5′-TACCTTGTTACGACTT-3′; *E. coli* bases 1,507–1,492) (Wilson et al. 1990). Alignment of the 16S rRNA genes and Rep protein sequences were accomplished using Clustal X (Thompson et al. 1997). Phylogenetic analyses were performed using PHYLIP (version 3.6; distributed by J. Felsenstein, University of Washington, Seattle, USA). The trees were constructed by the neighbor-joining method. Bootstrap analyses were performed with 100 re-sampled data sets.

### Plasmid curing

Elevated incubation temperature (up to 42 °C), ethidium bromide (500 μg/mL), sodium dodecylsulfate (SDS, 10 %) and acridine orange (500 μg/mL) were employed to eliminate the plasmids from their hosts.

### Comparisons of antibiotics and heavy metal ions resistance

Disc diffusion susceptibility testing method was used to determine antibiotics resistance profiles of the plasmid(s)-bearing isolates as described previously (Zhang et al. 2006). The 21 antibiotics (Ampicillin, Carbenicillin, Cephalothin, Chloramphenicol, Clindamycin, Colistin Sulphate, Cotrimoxazole, Erythromycin, Fusidic Acid, Gentamicin, Methicillin, Nalidixic Acid, Nitrofurantoin, Novobiocin, Penicillin G, Streptomycin, Sulphatriad, Sulphamethizole, Sulphamethoxzole, Tetracycline and Trimethoprim) were used in tests. Effects of heavy metal ions on the growth of plasmid(s)-bearing isolates were investigated using the method described previously (Zhang et al. 2006), the selected metals included Cd^2+^, Zn^2+^, Cu^2+^, Mn^2+^, Pb^2+^ (test concentrations were 0, 10, 25 and 50 μM in the growth medium), Hg^2+^ (test concentrations were 0, 10 and 25 μM) and Cr^6+^ (test concentrations were 0, 50, 100 and 150 μM).

### Nucleotide sequences accession numbers

The 16S rRNA gene sequences of the 45 plasmid(s)-bearing *V. cholerae* isolates were deposited in the GenBank under accession numbers from DQ440932 to DQ440976, The complete nucleotide sequences of pVCG1.1, pVCG1.2 and pVCG4.1 were deposited in GenBank database under accession No. DQ787203, DQ787204 and DQ787205, respectively.

## Results

### Plasmids screening and grouping

**Fig. 2:**
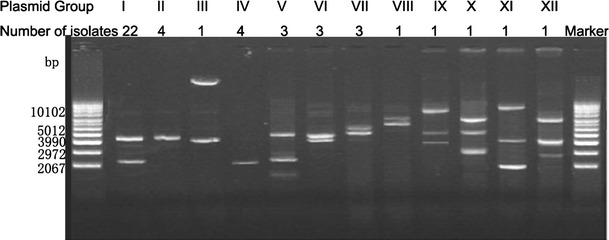
Plasmid profiles of the twelve groups of *Vibrio* isolates from Mai Po Nature Reserve, Hong Kong. *Lanes 1 and 14*, Supercoiled DNA ladder marker; plasmid profiles of Group I to Group XII and numbers of isolate in each group are indicated

The 45 plasmid(s)-bearing isolates harbored 1–3 plasmids. To determine their plasmid profiles, every single plasmid in the 45 plasmid(s)-bearing isolates was separately purified and their RFLP patterns were obtained using the enzymes of *Eco*RI and *Kpn*I (data not shown). Based on their plasmid profiles, these plasmid(s)-bearing isolates were divided into 12 groups, each group displayed its own unique plasmid profile (Fig. 2). Every plasmid(s)-bearing isolate was designated with the corresponding group number followed by the isolate number in this group, for example, isolate G1.1 is the first isolate in group I. Each plasmid was also named with the group number and the plasmid number (from large to small), for example, plasmid pVCG1.1 is the largest plasmid in group I and plasmid pVCG1.2 is the second largest plasmid in group I. Group I was the most abundant with 22 isolates, almost half of the 45 plasmid(s)-bearing isolates, this group harbored two plasmids with the size of about 2.4 and 4.4 kb (according to the supercoiled DNA markers). Groups II and IV had one plasmid with size of 4.4 and 2.2 kb, respectively, each group had four isolates. Groups V, VI and VII contained three isolates each. Other groups had one isolate only. 16S rRNA gene alignments showed all the plasmid(s)-bearing isolates belong to *Vibrio cholerae* (data not shown), a natural inhabitants of aquatic environments.

### Sequence analyses

**Fig. 3:**
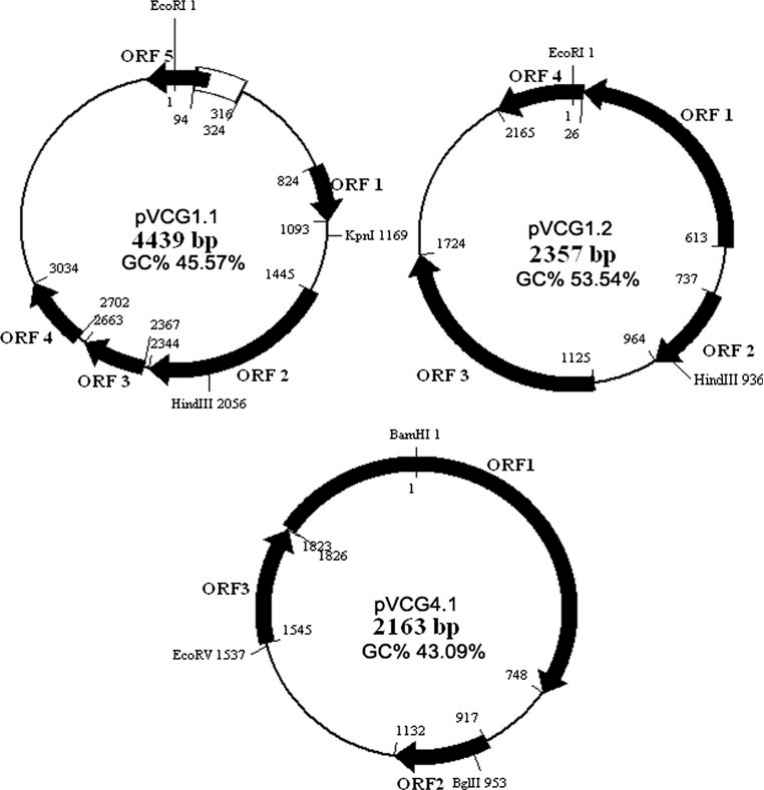
Physical maps of plasmid pVCG1.1, pVCG1.2 and pVCG4.1. Gene position and direction of transcription are indicated by *arrows*. The fragment (position 94–315) of plasmid pVCG1.1, which lacked in plasmid pVCG3.2 is indicated by *open box*

Complete sequences of the plasmids in groups I to IV (pVCG1.1, pVCG1.2, pVCG2.1, pVCG3.2 and pVCG4.1 but not the large plasmid pVCG3.1) were obtained, results showed pVCG1.1 and pVCG2.1 are identical, in pVCG3.2, only a 222 bp fragment (Fig. 3, position 94–316 in pVCG1.1) is lacked compared with its counterpart in groups I and II. Detailed sequence analysis revealed that there are two direct repeats (5′-CCCCTTAAC-3′, position 94–102 and 316–324) flanked this region, so one explanation is the 222 bp fragment was deleted through homologous recombination. However, this cannot exclude other mechanisms. The plasmid physical maps of pVCG1.1, pVCG1.2 and pVCG4.1 are shown in Fig. 3.

The circular plasmids pVCG1.1, pVCG1.2 and pVCG4.1 comprised of 4,439, 2,357 and 2,163 bp with the overall G+C content of 45.57, 53.54 and 43.09 %, respectively. The G+C contents of pVCG1.1 and pVCG4.1 are similar to, but that of pVCG1.2 is much higher than that of the two chromosomes (46.9 and 47.7 %, respectively) of *V. cholerae* (Heidelberg et al. 2000).

Plasmid pVCG1.1 has five ORFs, encoded the putative proteins of 89, 299, 98, 110 and 97 amino acids, respectively (Fig. 3), but they show no homologies to any known proteins. Most well studied plasmids encoded a replication initiation protein (Rep) which usually determine its replication type (Novick 1987). None of the potential proteins encoded by plasmid pVCG1.1 show any similarity to the known Rep proteins, suggesting that its replication mechanism may be different from that of the known plasmids.

Plasmid pVCG1.2 has four ORFs, encoded the putative proteins of 198, 75, 199 and 72 amino acids, respectively (Fig. 3). Blast searching revealed the predicted protein of ORF1 shows 39 % identity and 59 % similarity over 152 amino acids to the replication initiation protein (Proteins: AAG23805) of *Pseudomonas fluorescens* plasmid pAM10.6 (Peters et al. 2001). Proteins encoded by other ORFs showed no homology to any known proteins, and their functions are unknown.

Plasmid pVCG4.1 has three ORFs, encoding the putative proteins of 361, 71 and 92 amino acids, respectively (Fig. 3). The predicted protein of ORF1 has 37 % identity and 53 % similarity over 286 amino acids to the replication initiation protein CRI (Proteins: CAA32521) of *Enterobacteria* phage I2-2 (Stassen et al. 1992). Proteins encoded by the other two ORFs showed no homology to any known proteins.

**Fig. 4:**
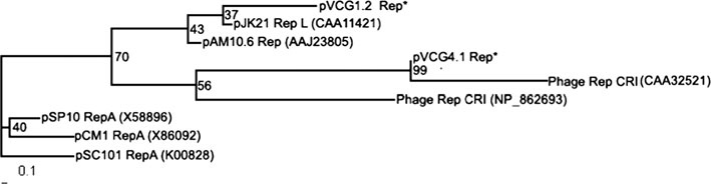
Phylogenetic relationship of plasmid replication initiation proteins of plasmids pVCG1.2 and pVCG4.1 and other referenced protein sequences. The tree was constructed by the neighbor-joining method with Rep protein sequence of plasmid pSC101 as root. *Asterisks* indicate the proteins in this study, and others are references

Since both pVCG1.2 and pVCG4.1 encoded a putative protein similar to the known Rep proteins of theta type replicons. They may belong to the theta type plasmids. The phylogenetic tree of their Rep proteins and those of other theta type replicons is shown in Fig. 4. Plasmids pVCG1.1 and pVCG1.2 co-exist in the same cell, according to incompatibility theory (Novick 1987), they should belong to different incompatibility groups, which in most cases are classified based on replication initiation proteins.

So far, including the three plasmids reported here, there are totally five completely sequenced *V. cholerae* plasmids in NCBI database of plasmid project under *V. cholerae* genome project (http://www.ncbi.nlm.nih.gov/sites/entrez?db=genomeprj&cmd=Retrieve&dopt=Overview&list_uids=30307). Previously we also identified and sequenced a *V. cholerae* plasmid, pVC, from marine environment (Zhang et al. 2007). Their main properties were compared in Table 1. Among these plasmids, only pTLC was isolated from a clinical strain, all others were from the marine environmental isolates. Plasmid pVCG1.2 has a much higher GC content, all other sequenced plasmids have a similar GC content to that of the *V. cholerae* chromosomes.

**Table 1:** *Vibrio cholerae* plasmids comparison

Plasmids	Accession no.	Size (bp)	GC content (%)	ORFs	Clinical/environmental
pTLC	AF052650	4,719	46.6	5	Clinical
pSIO1	AY876057	4,906	48.0	3	Environmental
pVC	AY423429	3,806	43.3	4	Environmental
pVCG1.1	DQ787203	4,439	45.6	5	Environmental
pVCG1.2	DQ787204	2,357	53.5	4	Environmental
pVCG4.1	DQ787205	2,163	43.1	3	Environmental

### Plasmids curing

To investigate their functions, different approaches (as described in “Materials and methods”) were used in an attempt to cure the plasmid(s) from their hosts in the 12 groups, but none succeeded.

### Comparisons of antibiotics and heavy metal ions resistance

Since isolates G1.1, G2.1 and G3.1 share a same plasmid, G3.1 also has another large plasmid pVCG3.1, their antibiotics and heavy metal ions resistance profiles were compared. Results showed isolates G1.1, G2.1 and G3.1 have the same performance to the 21 kinds of tested antibiotics (data not shown), within the tested concentrations, Cd^2+^, Zn^2+^, Cu^2+^, Mn^2+^, Pb^2+^ and Cr^6+^ have the same effect on the growth of isolates G1.1, G2.1 and G3.1 (data not shown), but for Hg^2+^, only isolate G3.1 showed tolerance to 10 μM Hg^2+^ (Table 2).

**Table 2:** Effects of Hg^2+^ concentrations on maximum biomass yield of isolates G1.1, G2.1 and G3.1

Isolates	Hg^2+^ concentrations (μM)		
	0	10	15
G1.1	0.767 ± 0.010	0.007 ± 0.001	0.007 ± 0.001
G2.1	0.631 ± 0.009	0.009 ± 0.002	0.011 ± 0.002
G3.1	0.692 ± 0.024	0.297 ± 0.136	0.011 ± 0.001

*SD* standard deviation of triplicate

## Discussion

In this study, we investigated the plasmids incidence and diversity in *Vibrio* community of Mai Po Nature Reserve, the incidence of plasmid was relatively high in the environmental *Vibrio* isolates, plasmid(s)-bearing isolates accounted for 45 % of the *Vibrio* isolates, and most (82.2 %) of them contained multiple plasmids, isolates containing 2 and 3 plasmids accounted for 66.7 and 15.6 %, respectively. The most abundant plasmid pVCG1.1 showed no homology to the known Rep proteins, and probably represents a novel plasmid, further studies will determine the *rep* gene and replication mechanism of this plasmid. This phenomenon is in agreement with several other reports (Dahlberg et al. 1997; Sobecky et al. 1997, 1998; Bidinost et al. 1999; Pan et al. 2010), which have showed that plasmids from marine bacteria have no detectable homology with plasmids from clinical isolates, indicating that bacterial isolates obtained from marine environment are a source of novel plasmids.

All attempts to cure the plasmids from their hosts were failed, probably due to the relatively high copy number of the plasmids similar to earlier work (Zhang et al. 2006; 2007). So far, the majority of bacteria adapted to survive and proliferate in the presence of mercury have been shown to reduce Hg^2+^ to its volatile form Hg° via the well-characterized *mer* operons (Rochelle et al. 1991; Silver and Walderhaug 1992; Jeffrey et al. 1996; Liebert et al. 1997). Mercury resistant genes were reported to be located on large plasmids (Summers and Silver 1972; Rani and Mahadevan 1994; Silver 1996). In addition, other mercury resistance mechanism such as efflux system in marine bacteria was reported to be plasmid-encoded (Reyes et al. 1999). Isolate G3.1 has a relatively large plasmid pVCG3.1, a relationship of tolerance to 10 μM Hg^2+^ of G3.1 and presence of plasmid pVCG3.1 may exist but need further direct evidence. Amplification of the possible *mer* operons in plasmid pVCG3.1 and G3.1 genomic DNA using the primers based on the reported *mer* operons did not confirm the results (data not shown).

In conclusion, *V. cholerae* community of Mai Po Nature Reserve, Hong Kong showed high incidence and diversity of plasmids.
